# Pathological significance and prognostic significance of FES expression in bladder cancer vary according to tumor grade

**DOI:** 10.1007/s00432-017-2524-1

**Published:** 2017-09-26

**Authors:** Akihiro Asai, Yasuyoshi Miyata, Kosuke Takehara, Shigeru Kanda, Shin-ichi Watanabe, Peter A. Greer, Hideki Sakai

**Affiliations:** 1 Department of Urology, Nagasaki University Graduate School of Biomedical Sciences, 1-7-1 Sakamoto, Nagasaki, 852-8501 Japan; 2 Division of Cancer Biology and Genetics, Department of Pathology and Molecular Medicine, Queen’s Cancer Research Institute, Queens University, Kingston, ON K7L 3N6 Canada

**Keywords:** FES, Grade, Cell proliferation, Invasion, Prognosis, Bladder cancer

## Abstract

**Purpose:**

The feline sarcoma oncogene protein (FES) is a non-receptor tyrosine kinase implicated in both oncogenesis and tumor suppression. Here, cancer cell lines and human tissues were employed to clarify the pathological and prognostic significance of FES in bladder cancer.

**Methods:**

The relationship between FES expression and cancer aggressiveness was investigated using 3 cell lines (T24: corresponding to grade 3, 5637: corresponding to grade 2, and RT4: corresponding to grade 1) and 203 tissues derived from human bladder malignancies. Proliferation, invasion, and migration of cancer cells were assessed following the knockdown (KD) of FES expression by the siRNA method. Relationships between FES expression and pathological features, aggressiveness, and outcome were investigated.

**Results:**

FES-KD inhibited the proliferation, migration, and invasion of T24 cells but not of RT4 cells and 5637 cells. Considering all patients, FES expression demonstrated a negative relationship with grade but no association with muscle invasion or cancer cell proliferation. However, it was positively correlated with pT stage and cell proliferation in high-grade tumors (*p* = 0.002); no such association was found for low-grade tumors. In addition, elevated FES expression was a negative prognostic indicator of metastasis after radical surgery for patients with high-grade tumors (*p* = 0.021) but not for those with low-grade malignancies.

**Conclusions:**

FES appeared to act as a suppressor of carcinogenesis, being associated with low tumor grade in the overall patient group. However, its expression correlated with cancer aggressiveness and poor outcome in high-grade bladder cancer. FES, therefore, represents a potential therapeutic target and useful prognostic factor for such patients.

## Introduction

Bladder cancer is a common malignancy, particularly in industrialized countries, and it exhibits a relatively high metastasis rate. This disease imposes a substantial psychological, physical, and economic burden, yet unfortunately, despite various multidisciplinary therapeutic approaches, the prognosis of patients with metastatic bladder cancer remains poor (Abufaraj et al. 2016). It is generally agreed that bladder cancer aggressiveness comprises pathological features such as muscle invasion and tumor grade. Therefore, understanding such pathological characteristics and their prognostic roles at a molecular level is essential for improving treatment and observation strategies for patients with this condition.

The feline sarcoma oncogene protein (FES) is a member of the FES/FES-related (FER) subfamily of non-receptor protein tyrosine kinases (Greer 2002). The original identification of *FES* as a retroviral oncogene underscores its potential role in cancer; however, understanding of its function has been complicated by subsequent research that has implicated FES in both tumor-promotive and tumor-suppressive roles (Bardelli et al. 2003; Delfino et al. 2006; Sangrar et al. 2005; Voisset et al. 2010; Zhang et al. 2011; Olvedy et al. 2017). In a prior study, we showed that FES downregulation inhibits the proliferation of renal cell carcinoma cells (Kanda et al. 2009). In addition, we previously reported that increased FES expression correlates with more aggressive disease and shortened recurrence-free survival periods after surgical resection (Miyata et al. 2012). On the other hand, kinase-inactivating mutations in the *FES* gene have been detected in colorectal cancer cells (Bardelli et al. 2003; Sangrar et al. 2005), and low or absent FES expression has been reported in colon cancer specimens compared with matched normal tissues (Kanda et al. 2009). In addition, tumor onset in the mouse mammary tumor virus–polyomavirus middle T transgenic mouse model of breast cancer was found to be accelerated as a result of *FES* knockout (Sangrar et al. 2005). Collectively, these observations suggest that FES may exert both tumorigenic and tumor-suppressive effects. To the best of our knowledge, the involvement of FES in bladder cancer has not been described thus far.

The present study was designed to determine the relationship between FES expression and bladder cancer aggressiveness, including malignant cell proliferation and invasion, in vivo and in vitro. The pathological and prognostic significance of FES expression was assessed in patients with bladder cancer, with particular attention to the effect of cancer grade on the relationship between FES levels and pathological characteristics.

## Materials and methods

### Cell culture and siRNA

Three human urothelial carcinoma cell lines, T24 (corresponding to grade 3), 5637 (grade 2), and RT4 (grade 1), were purchased from the American Type Culture Collection (Manassas, VA, USA). Cells were cultured in Dulbecco’s modified Eagle’s medium (DMEM) (Gibco/Thermo Fisher Scientific, Waltham, MA, USA) supplemented with 10% fetal bovine serum (FBS) and 50 µg/ml gentamicin (Gibco/Life Technologies) at 37 °C in a humidified atmosphere of 5% CO_2_ and 95% air. Each line was seeded at 5 × 10^5^ cells per 100-mm dish. After 24 h, cells were treated with jetPRIME transfection reagent (Polyplus-transfection Inc., New York, NY, USA) and 50-nM *FES* siRNA, according to the manufacturer’s protocol. FES expression was then assessed by western blotting. The specificity and reliability of these commercial agents were confirmed in our previous report (Mitsunari et al. 2016). In addition, non-specific effects were ruled out using non-specific control siRNA (Negative Control siRNA, Qiagen, Venlo, The Netherlands) according to our previous report (Mitsunari et al. 2016).

### Evaluation of proliferation and growth in cancer cell lines

Relative viable cell number was determined using the methylthiazolyltetrazolium (MTT) assay (Cell Proliferation Kit I (MTT); Roche, Basel, Switzerland). T24, 5637, and RT4 cells were placed in each well of a 96-well plate and allowed to adhere and spread for 24 h. The MTT labeling reagent was added to each well, and the cultures were incubated for 4 h at 37 °C. Solubilization solution was then added, and the cells were incubated in a humidified atmosphere overnight. Cell densities were determined by measuring absorbance at 550 nm.

### Evaluation of cell invasion and migration

Cells (0.3 × 10^6^) were incubated for 48 h in polycarbonate membrane inserts for use with a CytoSelect Cell Invasion Assay fluorometric kit (Cell Biolabs Inc., San Diego, CA, USA). Invasive cells having passed through the membrane were then lysed, and this lysate was transferred to a 96-well plate for the measurement of fluorescence (expressed in relative fluorescence units) in a plate reader at 480 nm.

Cells (2.1 × 10^4^) were seeded in a 2-well culture insert (ibidi GmbH, Martinsried, Germany) placed on the bottom of a 35-mm culture dish, and incubated for 24 h. A cell-free gap 500-μm wide was created between the two cell populations after removing the insert, and the medium was replaced with DMEM containing 10% FBS. T24 cells were incubated for 6 and 12 h, 5637 cells for 6 and 8 h, and RT4 cells for 24 and 48 h. Cell migration was subsequently evaluated by microscopically measuring the gap.

### Western blot analysis

Western blotting was performed as described previously (Mitsunari et al. 2016). Briefly, cultured cells were harvested by scraping and were lysed in ice-cold hypotonic cell lysis buffer containing protease inhibitors. Aliquots of total cellular lysate (40–50 μg/lane) were electrophoresed on sodium dodecyl sulfate–polyacrylamide gels and transferred to nitrocellulose membranes. After membranes were blocked with 5% non-fat milk in Tris-buffered saline containing 0.1% Tween 20 (TBST) for 1 h at 22 °C, each membrane was incubated with the primary antibody overnight at 4 °C. After three washes with TBST, membranes were incubated with the appropriate secondary antibody for 1 h at room temperature. Specific protein bands were visualized using ECL Prime (GE Healthcare, Little Chalfont, UK).

### Patients

A total of 203 bladder cancer specimens obtained from consecutive cases by transurethral resection (TUR) or radical cystectomy between 1995 and 2006 at Nagasaki University Hospital were examined. Only patients diagnosed by biopsy were excluded from this study. Nineteen specimens were excluded from further analysis as they contained fewer than 300 cancer cells. We also excluded patients who had received neoadjuvant therapy, including chemotherapy or radiotherapy. All patients underwent cystoscopy, ultrasonography, computed tomography (CT), and/or magnetic resonance imaging of the pelvis to evaluate preoperative metastatic status. In addition, CT of the lungs or brain, drip infusion pyelography, and bone scanning were performed when deemed necessary. Specimens obtained during surgery were used for pathological diagnosis, and staging was assessed according to the 2009 tumor-node-metastasis classification system. As a general rule, at our institution, radical surgery is contraindicated for patients with metastasis and tumors of stage T4. In addition, patients with such advanced disease often receive neoadjuvant therapy. Therefore, patients with cancer of this stage and/or metastasis were also excluded. Thus, 203 patients were enrolled in this study after application of the above criteria. Although we recommended adjuvant treatment, including postoperative intravesical therapy, 60 patients (29.6%) chose not to receive this treatment. Of the other patients, 133 (65.5%) and 10 (4.9%) received intravesical therapy and systemic chemotherapy, respectively. As controls, cancer-free bladder tissues (*n* = 20) were obtained by TUR or biopsy. Our study protocol conformed to the rules of the Human Ethics Review Committee of Nagasaki University Graduate School of Medicine.

### Immunohistochemical staining and evaluation of the samples

Bladder tissue sections (5-µm thick) were deparaffinized in xylene and rehydrated using a graded ethanol series. Antigen retrieval was performed at 95 °C for 40 min in 0.01-M sodium citrate buffer (pH 6.0). Sections were then immersed in 3% hydrogen peroxide for 30 min to quench endogenous peroxidase activity, and incubated overnight at 4 °C with the primary antibody (anti-FES; C-19; Santa Cruz Biotechnology, Santa Cruz, CA, USA). Subsequently, the sections were washed in 0.05% Tween 20 in phosphate-buffered saline, and incubated with peroxidase using an LSAB™ + kit (Dako Corp., Carpinteria, CA, USA) according to the manufacturer’s instructions. The peroxidase reaction was visualized with a liquid 3,3′-diaminobenzidine tetrahydrochloride substrate kit (Zymed Laboratories Inc., South San Francisco, CA, USA). Sections were counterstained with hematoxylin, dehydrated through graded alcohol solutions, and cleared using xylene, before being mounted with Poly-Mount (Polysciences, Inc., Warrington, PA, USA). The specificity and sensitivity of the FES antibody used was verified in our previous report (Miyata et al. 2012). In short, normal kidney specimens confirmed in preliminary studies to be immunoreactive with this antibody were used as positive controls. Consecutive sections from each sample processed as above but without the addition of the primary antibody were used as negative controls. Positive and negative controls were included in each batch of samples. In addition, to confirm the specificity of the primary antibody, we also conducted a competition study using a blocking peptide (sc-7670 P, Santa Cruz Biotechnology) according to a previous report (Miyata et al. 2012). Furthermore, the specificity of this primary antibody for human c-Fes protein was again confirmed by comparing its expression by immunoblotting with that by immunohistochemistry, as described previously by our group (Ohba et al. 2011). The proliferation index (PI) was defined as the percentage of Ki-67-positive cancer cells detected using an anti-Ki-67 antibody (Dako Corp., Glostrup, Denmark). Detailed methods, including a description of the positive control, are given in our previous report (Miyata et al. 2014).

FES expression was measured semi-quantitatively using the method described by Zoubeidi et al. (2009). Staining intensity was graded as negative, weak, moderate, or strong. For the human tissues examined here, expression level was quantified using the immunoreactivity score (IRS), where IRS = staining intensity × percentage of positive cells. Staining intensity was defined as follows: 0, negative; 1, weak; 2, moderate; and 3, strong; while the percentage of positive cells was scored in the following way: 0, 0–10%; 1, 11–20%; 2, 21–40%; and 3, 41–100%. Three investigators (Y.M., S.K., and S.W.) independently performed these semi-quantitative analyses. When the conclusions of the three investigators differed, a final decision was reached by majority rule. For statistical analysis, patients were divided into two groups based on IRSs—“negative” and “positive”—the positive group comprising those with an IRS above the median value. Semi-quantitative analysis was performed on at least 500 cancer cells in 3–6 visual fields per section. Each field was also examined at 200× magnification using an E-400 microscope (Nikon, Tokyo, Japan), and representative images were taken with a digital camera (DU100; Nikon) and subjected to image analysis (Win ROOF version 5.0; MITANI Corp., Fukui, Japan). FES expression in cancerous stromal tissues was judged positive when the intensity and area of staining were similar to or higher than those observed in normal stromal tissues.

### Statistical analysis

Normality was evaluated by normal distribution and histograms for each variable, and results are expressed as medians; interquartile range (IQR) and/or mean ± SDs. The Mann–Whitney *U* test and Student’s *t* test were used for comparisons involving non-parametric and parametric continuous variables, respectively. The Scheffé test was employed for multiple comparisons. The Chi-squared test was used to compare categorical data. Differences in survival were assessed using Kaplan–Meier curves, the log-rank test, and Cox regression analysis, and are expressed using hazard ratios (HRs), 95% confidential intervals (95% CIs), and *p* values, respectively. All statistical analyses were two-sided and performed in StatView for Windows (version 5.0; Abacus Concepts, Inc., Berkeley, CA, USA); *p* < 0.05 was considered to represent statistical significance.

## Results

### Proliferation and FES expression in cancer cell lines

**Fig. 1:**
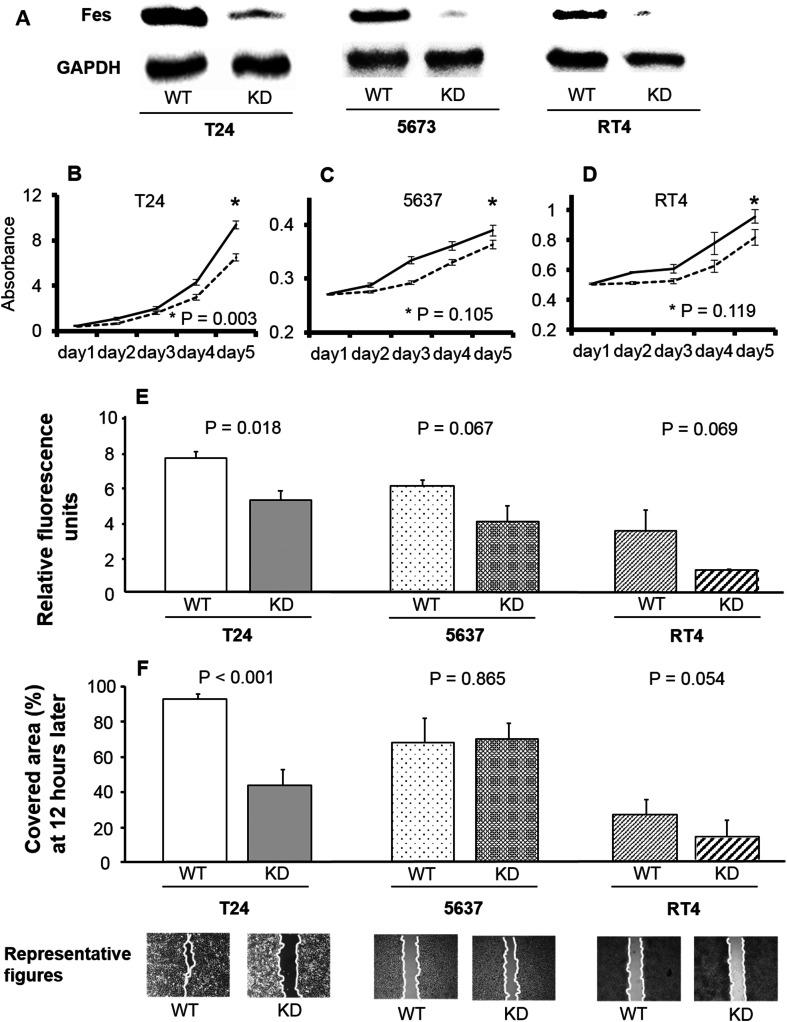
Pathological roles of FES in various bladder cancer cell lines. **a** FES expression in non-transfected and FES-KD cells. In all three lines, FES expression was remarkably decreased following KD. **b**–**d** Growth curves of cancer cells of each line. Cell proliferation was inhibited by KD of FES expression in T24 cells (**b**; corresponding to grade 3) (*p* < 0.001); however, no such effect was detected using 5637 cells (**c**; grade 2) or RT4 cells (**d**; grade 1). **e** Relative fluorescence representative of invasive cell number was lower following KD of FES expression in T24 but not 5637 or RT4 cells. **f** Similar results were obtained with a gap-closure assay used to evaluate cell migration. Briefly, inhibition of cell migration by FES-KD was detected in T24 (*p* < 0.001) but not 5637 or RT4 cells. Representative figures of the scratch assay in these cell lines 12 h later are also shown

FES expression in non-transfected and FES-KD cells of each line is shown in Fig. 1a. FES expression was clearly decreased by KD in all cancer cell lines. The proliferation of T24 cells was significantly inhibited by KD of FES (*p* < 0.001, Fig. 1b). In contrast, the proliferation of 5637 cells or RT4 cells was not significantly influenced by FES-KD (*p* = 0.105 and 0.119, respectively; Fig. 1c, d).

As shown in Fig. 1e, invasion was significantly suppressed by FES-KD in T24 cells (*p* = 0.018). Invasiveness of FES-KD 5637 and RT4 cells tended to be lower than that of the non-transfected controls; however, these differences were not statistically significant (*p* = 0.067 and 0.069, respectively).

With regard to the migration of cancer cell lines, representative examples for each cell line are shown in Fig. 1f. Finally, as shown in Fig. 1f, the area of the cell-free gap covered by FES-KD T24 cells was significantly reduced compared to that occupied by untreated controls (*p* < 0.001). In contrast, no such difference was noted in relation to FES-KD in 5637 cells (*p* = 0.865). The area covered by FES-KD RT4 cells was smaller than that observed in the corresponding control group but not to a significant extent (*p* = 0.054).

### FES expression and its clinical significance in human cancer tissues

**Fig. 2:**
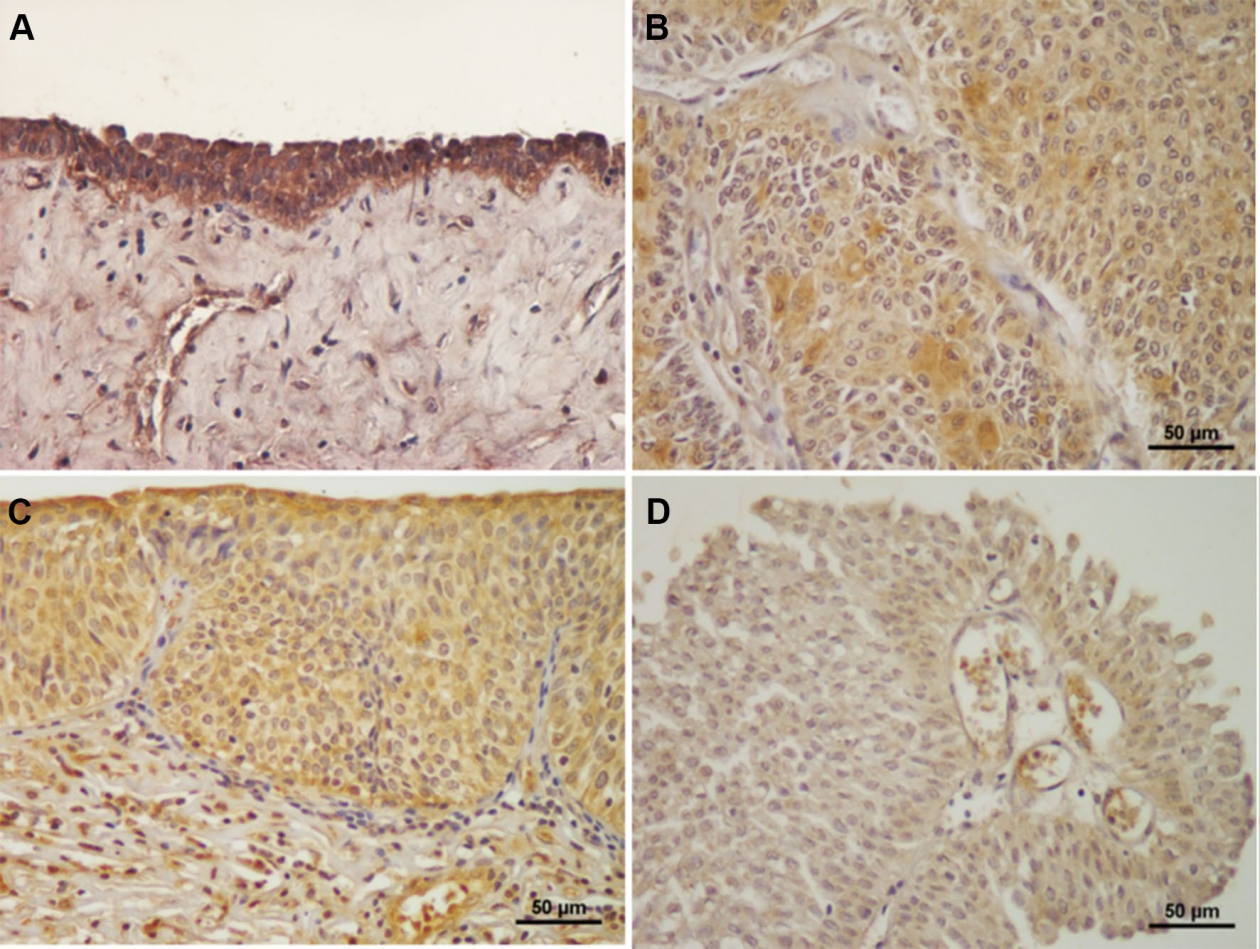
FES expression in human bladder cancer tissues. Almost all normal urothelial cells stained positive for FES (**a**). In contrast, although weak FES staining was often observed in cancer cells, moderate expression was only occasionally detected, and strong expression was scarce (**b**). In addition to cancer cells, FES expression was detected in stromal-infiltrating, endothelial, and hematopoietic cells (**c**). Finally, **b** and **c** was judged as “positive”, because their FES IRSs were 4 and 3, respectively (positive expression is defined as IRS > 2). In contrast, a reported example (**d**) was judged as “negative” (IRS = 1)

Figure 2a shows a representative normal human tissue section containing FES-expressing urothelial cells. The expression of FES was principally detected in the cytoplasm, and was strong in almost all normal cells. In contrast, although FES immunostaining was often observed in bladder cancer cells, strong expression was only occasionally evident (Fig. 2b). In addition, positive FES immunostaining was noted in fibroblast-like, endothelial, hematopoietic, and stromal-infiltrating cells (Fig. 2c).

FES IRSs of cancerous specimens (median = 2.0, IQR = 1.5–3.0, mean ± SD = 2.51 ± 1.58) were significantly lower (*p* < 0.001) than those of normal urothelial tissues (4.5, 3–5, and 4.25 ± 1.55). Relationships between cancer cell FES expression and clinicopathological features of malignancies are shown in Table 1. FES IRS in muscle-invasive bladder cancer (MIBC) (2.5, 1.5–3.0, and 2.83 ± 1.89) tended to be higher than in non-MIBC (2.0, 1.5–3.0, and 2.44 ± 1.5), albeit not significantly so (*p* = 0.109). In contrast, FES expression negatively correlated with grade (low grade; 2.5, 1.5–4.0, and 2.83 ± 1.67 versus high grade; 1.5, 1.5–2.5, 2.18 ± 1.40; *p* = 0.005). No significant association was established for FES expression with age at diagnosis or sex.

**Table 1:** FES expression and clinicopathological features

	Patients *N* (%)	Immunoreactive score of FES		*p* value
		Median, IQR	Mean/SD	
Age at diagnosis				0.291
Median or less	102 (50.2)	2.5, 1.5–3.0	2.50/1.47	
Over median	101 (49.8)	2.5, 1.5–2.5	2.29/1.45	
Sex				0.131
Male	166 (81.8)	2.0, 1.5–3.0	2.57/1.61	
Female	37 (18.2)	2.0, 1.0–2.5	2.22/1.40	
pT stage				0.039*
Ta	68 (33.5)	2.5, 1.0–4.0	2.76/1.74	
T1	100 (49.3)	2.0, 1.5–3.0	2.17/1.21	
T2	21 (10.3)	2.5, 1.5–3.0	2.41/1.26	
T3	14 (6.9)	3.5, 1.0–6.0	3.46/2.48	
Muscle invasion				0.109
Absence: Ta + 1	168 (82.8)	2.0, 1.5–3.0	2.44/1.50	
Presence: T2 + 3	35 (17.2)	2.5, 1.5–3.0	2.83/1.89	
Grade				0.005
Low	102 (50.2)	2.5, 1.5–4.0	2.83/1.67	
High	101 (49.8)	1.5, 1.5–2.5	2.18/1.40	

Data of immunoreactive score were showed as median, interquartile range (IQR), and mean/SD

*Significant value was detected between pT1 versus pT3 only

**Fig. 3:**
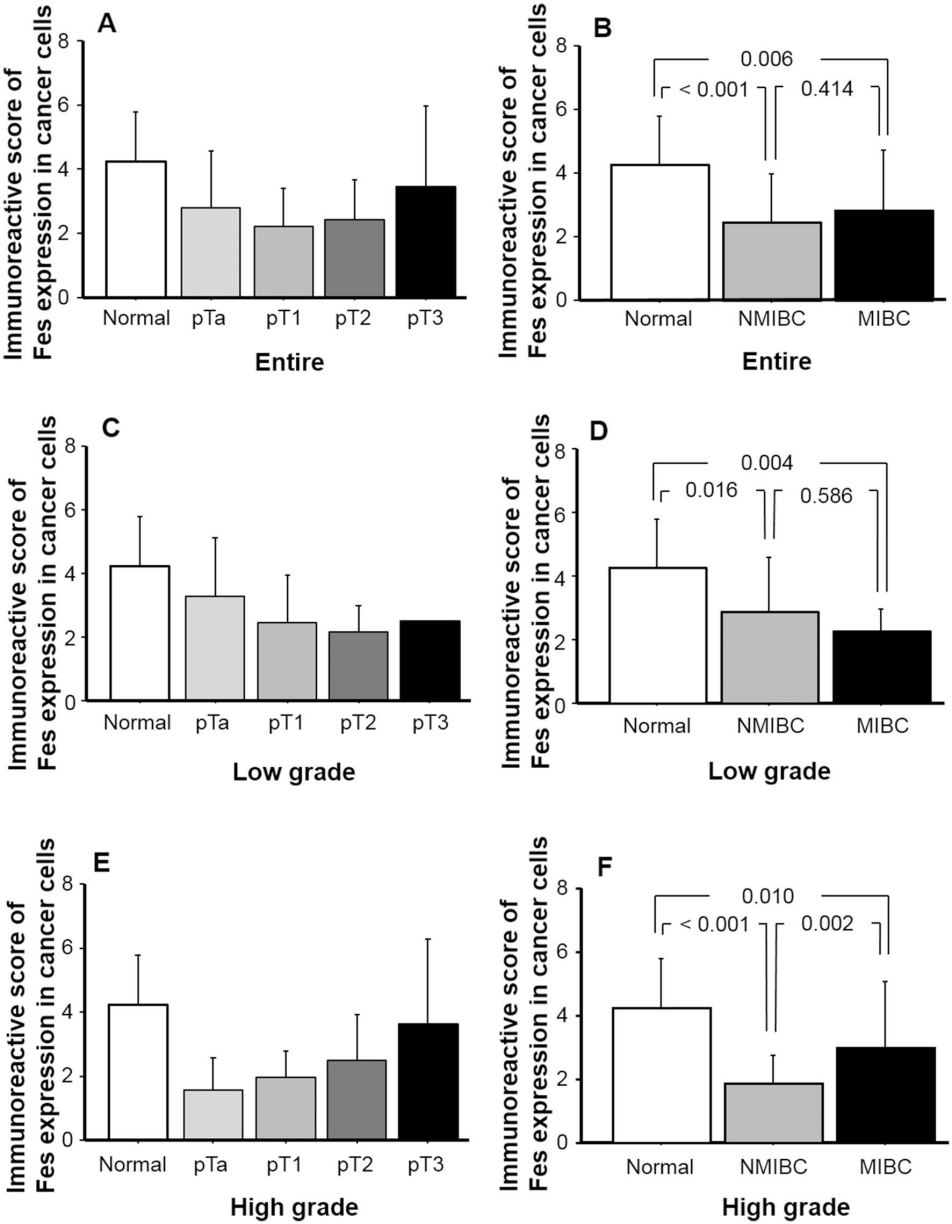
FES expression and cancer cell invasion in human bladder cancer tissues. FES IRS of normal and cancerous urothelium among all patients (**a**, **b**) and those with low-grade tumors (**c**, **d**) and high-grade tumors (**e**, **f**). The distribution of IRSs among pT stages differed between the low- and high-grade tumor groups (**c**, **d**). Considering all patients together, FES IRSs of NMIBC and MIBC tissues were significantly lower (*p* < 0.001 and 0.006, respectively) than those recorded for normal epithelia (**b**), and similar results were obtained when patients were categorized into low-grade (**d**) and high-grade groups (**f**). However, although in the low-grade group, FES IRS did not significantly differ between NMIBC and MIBC specimens (**d**), the latter exhibited significantly higher scores (*p* = 0.002) than the former in the high-grade group (**f**)

Figure 3 depicts FES IRS in normal urothelial tissues and according to pT stage and muscle invasion status. As shown in Fig. 3a, FES expression was higher in normal urothelium than in pT1 specimens, compared to which pT3 tissues exhibited higher levels. Moreover, multiple comparison tests revealed that FES IRSs in NMIBC and MIBC were significantly lower (*p* < 0.001 and 0.006, respectively) than those of normal epithelial samples. No significant difference was identified between NMIBC and MIBC in this respect (*p* = 0.414; Fig. 3b). Based on our in vitro results, we analyzed the relationship between FES IRS and invasiveness in low- and high-grade cancers separately. In the low-grade group, although decreases in FES IRS were noted at each subsequent pT stage up to pT2 (Fig. 3c), there was no significant difference in this regard between NMIBC and MIBC (*p* = 0.586; Fig. 3d). In contrast, among high-grade tumors, FES IRS demonstrated consistent increases from stage pTa to pT3/4 (Fig. 3e), and was significantly higher in MIBC than NMIBC (*p* = 0.002; Fig. 3f).

**Fig. 4:**
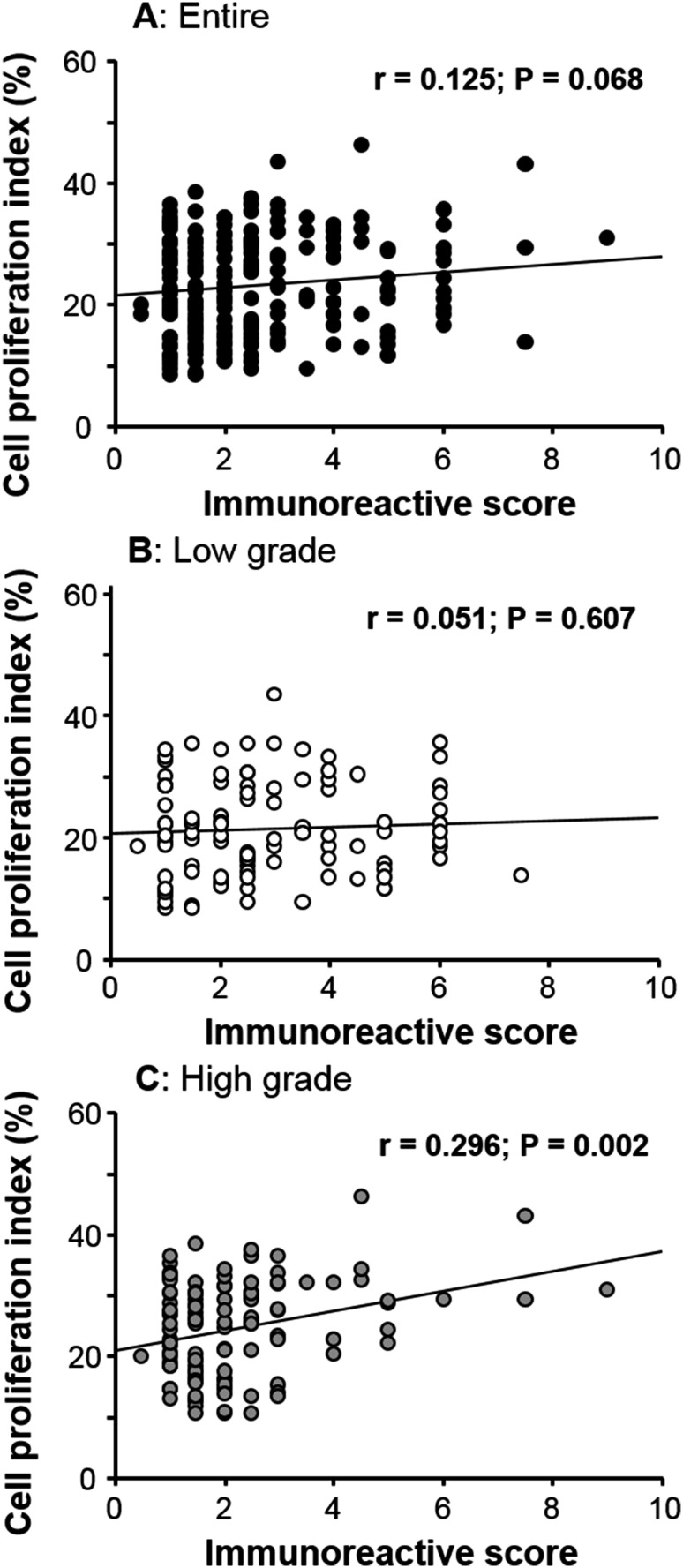
Correlations between FES expression and cancer cell proliferation. No significant correlation was established for the total patient (**a**) and low-grade tumor groups (**b**). However, considering only patients with high-grade tumors, a positive correlation was detected (*r* = 0.296; *p* = 0.002)

Regression analysis suggested a positive correlation between FES expression and PI in human cancer tissues when all patients were considered together; however, this relationship was not statistically significant (*r* = 0.125, *p* = 0.068; Fig. 4a). While no association was evident between these factors among low-grade tumor samples (*r* = 0.051, *p* = 0.607; Fig. 4b), a significant positive correlation was identified in the high-grade tumor group (*r* = 0.296, *p* = 0.002; Fig. 4c). The summary of pathological roles of FES expression according to malignant potential is presented in Table 2.

**Table 2:** Summary of pathological effects of FES expression

	Malignant potential	
	Low (grade 1 and 2)	Low (grade 1 and 2)
Cell lines		
Proliferation	Not significant	Stimulates
Migration	Not significant	Stimulates
Invasion	Not significant	Stimulates
Human tissues		
Proliferation	Not significant	Stimulates
Invasion	Not significant	Stimulates

### Survival analyses

**Fig. 5:**
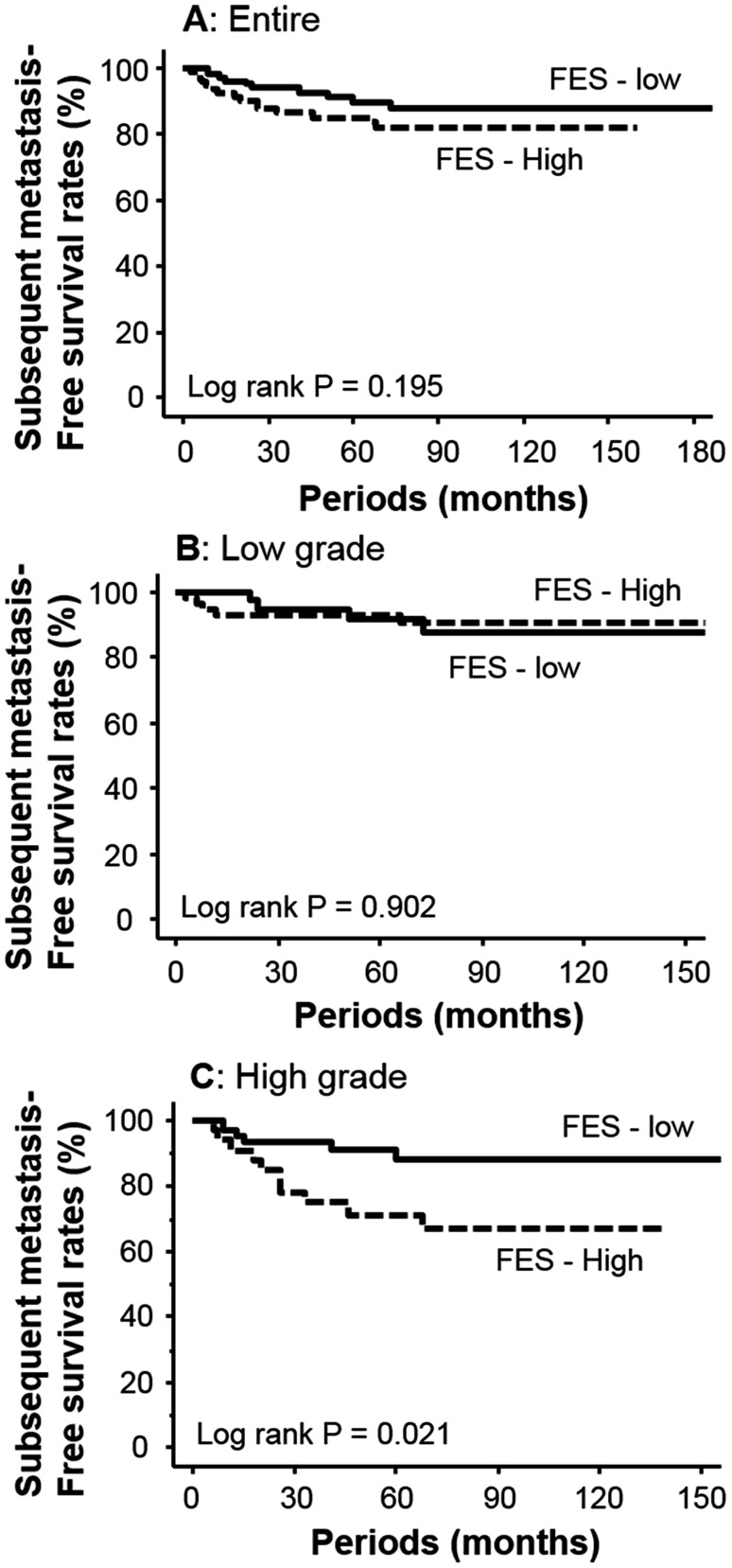
Kaplan–Meier survival curves for metastasis-free survival after radical surgery. FES expression was not associated with outcome in the total patient (**a**) or low-grade tumor groups (**b**). For patients with high-grade tumors, elevated FES level was identified as a negative prognostic factor (*p* = 0.021)

In this study, 143 patients (70.4%) received adjuvant therapy, which did not significantly influence FES expression (*p* = 0.193). On the basis of the median levels of IRS, patients with over 2 IRS are recognized as “positive” for FES expression. Kaplan–Meier survival curves suggested that FES expression was not associated with metastasis after radical surgery (*p* = 0.195, Fig. 5a). However, in subgroup analyses based on tumor grade (Fig. 5b, c), elevated FES expression was associated with a lower metastasis-free survival rate for patients with high-grade cancer (*p* = 0.021; Fig. 5c). On the other hand, among those with low-grade tumors, FES expression exhibited no relationship with subsequent metastasis (*p* = 0.902, Fig. 5b). In a similar analysis, FES expression was not found to be significantly associated with overall survival in the total patient group (*p* = 0.508). In addition, no such relationship was established in either the low- (*p* = 0.763) or high-grade subgroups (*p* = 0.172). A multivariate model including all clinicopathological features identified muscle invasion (pT2 and 3; HR = 5.4, 95% CI = 2.24–13.03; *p* < 0.001) but not cancer cell FES expression (HR = 1.3, 95% CI = 0.52–2.97; *p* = 0.617) as an independent predictor of subsequent metastasis.

## Discussion

The previous in vivo and in vitro studies have suggested that FES can exert both tumor-stimulatory and tumor-suppressive effects on cancer cells, according to the type of malignancy in question (Delfino et al. 2006; Kanda et al. 2009; Miyata et al. 2012; Sangrar et al. 2005). In the present investigation, we initially suspected that FES was acting as a tumor suppressor, because its expression in cancer cells was significantly lower than in normal urothelial cells. The negative association that we established between FES expression and tumor grade supported this hypothesis. However, FES levels tended to positively correlate with cancer cell proliferation and invasion. Thus, this protein appeared to be simultaneously anti- and pro-carcinogenic in the context of bladder cancer. We, therefore, attempted to identify the variable explaining these contrasting activities, eventually finding them to vary according to tumor grade. Briefly, FES expression was found to be positively associated with cancer cell proliferation and invasion among patients with high-grade tumors but not among those with low-grade malignancies. In addition, results consistent with this observation were obtained using T24 (corresponding to grade 3) and RT4 cells (grade 1). On the basis of these results, we propose that the pathological significance of bladder cancer cell FES expression depends on tumor grade. This phenomenon might partly explain the multiple functions of FES in this malignancy. Meanwhile, a recent study demonstrated that FES activity was required for Flt3 function in acute myelogenous leukemia (AML), and in fact, dual inhibition of both Flt3 and FES might provide a therapeutic advantage for the treatment (Weir et al. 2017). In addition, other investigators reported that microRNA-125b regulates AML cell differentiation by directly targeting FES (Hu et al. 2017). However, such co-factors often play pathological roles according to malignant potential, such as grade and prognosis, in various malignancies (Leick et al. 2017; Luo et al. 2017). These facts indicate a possibility that unknown co-factors and/or regulators of FES activity affect cell proliferation and invasion in only high-grade bladder cancer.

To address the fact that FES expression was lower in cancerous tissue than in normal specimens and FES IRS was significantly reduced in high-grade compared to low-grade tumors, we turned our attention to the physiological roles of this protein in the normal urothelium of the urinary bladder. This organ stores urine for long periods of time and undergoes repeated expansion and contraction, requiring the maintenance and dynamic regulation of cell–cell and cell–matrix adhesion in the urothelium. Among the various adhesion molecules involved, those of the catenin/cadherin and integrin systems are strongly expressed in normal urothelium (Bryan et al. 2010; Erickson et al. 2008). Knockout-mouse phenotypes have implicated FES in cell–cell and cell–matrix adhesion of hematopoietic cells, such as leukocytes, macrophages, and mast cells (Parsons et al. 2006, 2007; Smith 2010). In addition, a similar function in cell adhesion has been reported in breast epithelium (Truesdell et al. 2009). While the specific molecular events behind the participation of FES in these processes are not fully understood, the closely related FER kinase has been mechanistically linked to the regulation of adherens junctions (Xu et al. 2004). We, therefore, speculate that carcinogenesis disturbs the cell–cell and cell–matrix interactions in which FES is involved in normal urinary bladder urothelium, leading to reduced FES expression in cancer cells, especially those of high-grade tumors.

This model may be considered inconsistent with the fact that FES expression was found to be positively associated with cancer aggressiveness. However, we also propose that the pathological influences of FES in the context of cell–cell and cell–matrix interactions are different from those in the absence of these processes. In fact, the biological activities of this enzyme are regulated by cell adhesion- and cell junction-related factors (Naba et al. 2008; Smith 2010). In short, loss of cell contact- and adherence junction-associated signaling due to increased malignancy may alter the pathological roles of FES in bladder cancer. Besides, FES expression in cancer cells may be increased by changes in the surrounding microenvironment. The pathogenic effects of FES and the regulation of cell adhesion molecules in bladder cancer are complex and vary according to such local changes (Elsamman et al. 2006; Izuhara et al. 1996; Mialhe et al. 1997). Furthermore, our results showed that FES was expressed by various types of stromal-infiltrating and endothelial cells, supporting this hypothesis.

A previous report demonstrated FES expression to be associated with outcome in prostate cancer patients who had undergone radical surgery (Miyata et al. 2012). However, the prognostic utility of FES levels in bladder cancer patients has not been examined. That increased FES expression was a significant negative prognostic factor for metastasis after radical surgery for patients with high- but not low-grade bladder cancer was a key result of the present study. We speculate that the significance of FES expression in cancer prognosis can be explained by its grade-dependent activities. In summary, greater aggressiveness, including cancer cell proliferation and invasion, is the cause of the discrepancy between low- and high-grade tumor characteristics.

The current investigation was limited by the relatively small number of patients with MIBC compared to the number of those with NMIBC, owing to the use of consecutive sampling. However, this does suggest that selection bias was minimal. A further potential source of concern was that FES expression in cancer tissues was assessed using a semi-quantitative method. This was addressed by independent evaluation of specimens by three investigators, whose verdicts agreed in over 75% of cases. Thus, we believe that methodological bias was negligible in this study.

In conclusion, the pathological and prognostic significance of FES expression depended on tumor grade in patients with bladder cancer. In certain contexts, FES exerts significant pathogenic effects that manifest as increased cancer malignancy. In addition, elevated FES levels were associated with greater aggressiveness and worse outcome in terms of subsequent metastasis in patients with high- but not low-grade bladder cancer. Our results identify FES as a potential therapeutic target and a useful prognostic factor for patients with high-grade bladder cancer. It is possible that the role of FES during the initial carcinogenesis in normal epithelium differs from that in cancer aggressiveness in later stages of the disease. Furthermore, key regulators of FES-related activities may be stimulated or inhibited in high-grade cancers. Finally, we emphasize that understanding such complex regulative mechanisms of FES is important to discuss and improve observation and treatment strategies. Therefore, wider and more detailed investigations are necessary for a comprehensive understanding of the pathological activities of FES and their regulation in bladder cancer.

## References

[CR1] Abufaraj M et al (2016) Management of muscle invasive, locally advanced and metastatic urothelial carcinoma of the bladder: a literature review with emphasis on the role of surgery. Transl Androl Urol 5:735–744 27785430 10.21037/tau.2016.08.23 PMC5071186

[CR2] Bardelli A et al (2003) Mutational analysis of the tyrosine kinome in colorectal cancers. Science 300:949 12738854 10.1126/science.1082596

[CR3] Bryan RT et al (2010) Cadherin switching and bladder cancer. J Urol 184:423–431. doi:10.1016/j.juro.2010.04.016 20620393 10.1016/j.juro.2010.04.016

[CR4] Delfino FJ et al (2006) A growth-suppressive function for the c-fes protein-tyrosine kinase in colorectal cancer. J Biol Chem 281:8829–8835 16455651 10.1074/jbc.M507331200

[CR5] Elsamman E et al (2006) Differences in gene expression between noninvasive and invasive transitional cell carcinoma of the human bladder using complementary deoxyribonucleic acid microarray: preliminary results. Urol Oncol 24:109–115 16520272 10.1016/j.urolonc.2005.07.011

[CR6] Erickson DR et al (2008) Differentiation associated changes in gene expression profiles of interstitial cystitis and control urothelial cells. J Urol 180:2681–2687. doi:10.1016/j.juro.2008.08.007 18951569 10.1016/j.juro.2008.08.007

[CR7] Greer P (2002) Closing in on the biological functions of Fps/Fes and Fer. Nat Rev Mol Cell Biol 3:278–289 11994747 10.1038/nrm783

[CR8] Hu J et al (2017) MicroRNA-125b inhibits AML cells differentiation by directly targeting FES. Gene 620:1–9. doi:10.1016/j.gene.2017.04.002 28389358 10.1016/j.gene.2017.04.002

[CR9] Izuhara K et al (1996) Interleukin-4 induces association of the c-fes proto-oncogene product with phosphatidylinositol-3 kinase. Blood 88:3910–3918 8916957

[CR10] Kanda S et al (2009) Downregulation of the c-Fes protein-tyrosine kinase inhibits the proliferation of human renal carcinoma cells. Int J Oncol 34:89–96 19082481 PMC2802829

[CR11] Leick MB et al (2017) The future of targeting FLT3 activation in AML. Curr Hematol Malig Rep 12:153–167. doi:10.1007/s11899-017-0381-2 28421420 10.1007/s11899-017-0381-2

[CR12] Luo Y et al (2017) Elevated microRNA-125b levels predict a worse prognosis in HER2-positive breast cancer patients. Oncol Lett 13:867–874. doi:10.3892/ol.2016.5482 28356971 10.3892/ol.2016.5482 PMC5351301

[CR13] Mialhe A et al (1997) Expression of E-cadherin and alpha-, beta- and gamma-catenins in human bladder carcinomas: are they good prognostic factors? Invasion Metastasis 17:124–137 9702939

[CR14] Mitsunari K et al (2016) Human antigen R is positively associated with malignant aggressiveness via upregulation of cell proliferation, migration, and vascular endothelial growth factors and cyclooxygenase-2 in prostate cancer. Transl Res 175:11–128. doi:10.1016/j.trsl.2016.04.002 10.1016/j.trsl.2016.04.002 27140699

[CR15] Miyata Y et al (2012) Pathological significance and predictive value for biochemical recurrence of c-Fes expression in prostate cancer. Prostate 72:201–208. doi:10.1002/pros.21422 21563194 10.1002/pros.21422

[CR16] Miyata Y et al (2014) Heme oxygenase-1 expression is associated with tumor aggressiveness and outcomes in patients with bladder cancer: a correlation with smoking intensity. Transl Res 164:468–476. doi:10.1016/j.trsl.2014.06.010 25063314 10.1016/j.trsl.2014.06.010

[CR17] Naba A et al (2008) Spatial recruitment and activation of the Fes kinase by ezrin promotes HGF-induced cell scattering. EMBO J 27:38–50 18046454 10.1038/sj.emboj.7601943 PMC2206125

[CR18] Ohba K et al (2011) Clinical significance and predictive value of prostaglandin E2 receptors (EPR) 1–4 in patients with renal cell carcinoma. Anticancer Res 31:597–605 21378344

[CR19] Olvedy M et al (2017) Comparative oncogenomics identifies tyrosine kinase FES as a tumor suppressor in melanoma. J Clin Investig 127:2310–2325. doi:10.1172/JCI91291 28463229 10.1172/JCI91291 PMC5451227

[CR20] Parsons SA et al (2006) The Fps/Fes kinase regulates the inflammatory response to endotoxin through down-regulation of TLR4, NF-kappaB activation, and TNF-alpha secretion in macrophages. J Leukoc Biol 80:1522–1528 16959897 10.1189/jlb.0506350

[CR21] Parsons SA et al (2007) The Fps/Fes kinase regulates leucocyte recruitment and extravasation during inflammation. Immunology 122:542–550 17627769 10.1111/j.1365-2567.2007.02670.x PMC2228385

[CR22] Sangrar W et al (2005) An identity crisis for Fps/Fes: oncogene or tumor suppressor? Cancer Res 65:3322–3518 10.1158/0008-5472.CAN-04-3468 15867340

[CR23] Smith JA (2010) Fps/Fes protein-tyrosine kinase regulates mast cell adhesion and migration downstream of Kit and beta1 integrin receptors. Cell Signal 22:427–436. doi:10.1016/j.cellsig.2009.10.014 19892014 10.1016/j.cellsig.2009.10.014

[CR24] Truesdell PF et al (2009) Fps/Fes knockout mice display a lactation defect and the fps/fes tyrosine kinase is a component of E-cadherin-based adherens junctions in breast epithelial cells during lactation. Exp Cell Res 315:2929–2940. doi:10.1016/j.yexcr.2009.08.021 19732771 10.1016/j.yexcr.2009.08.021

[CR25] Voisset E et al (2010) FES kinases are required for oncogenic FLT3 signaling. Leukemia 2:721–728. doi:10.1038/leu.2009.301 10.1038/leu.2009.301 20111072

[CR26] Weir MC et al (2017) Dual inhibition of FES and Flt3 tyrosine kinases potently inhibits Flt3-ITD + AML cell growth. PLoS One 12:e0181178. doi:10.1371/journal.pone.0181178.eCollection 28727840 10.1371/journal.pone.0181178 PMC5519068

[CR27] Xu G et al (2004) Continuous association of cadherin with beta-catenin requires the non-receptor tyrosine-kinase Fer. J Cell Sci 117:3207–3219 15226396 10.1242/jcs.01174

[CR28] Zhang S et al (2011) Fes tyrosine kinase expression in the tumor niche correlates with enhanced tumor growth, angiogenesis, circulating tumor cells, metastasis and infiltrating macrophages. Cancer Res 71:1465–1473. doi:10.1158/0008-5472 21159660 10.1158/0008-5472.CAN-10-3757 PMC3041852

[CR29] Zoubeidi A et al (2009) The fer tyrosine kinase cooperates with interleukin-6 to activate signal transducer and activator of transcription 3 and promote human prostate cancer cell growth. Mol Cancer Res 7:142–155. doi:10.1158/1541-7786 19147545 10.1158/1541-7786.MCR-08-0117

